# Very late onset LASIK flap Acremonium fungal keratitis confirmed by metagenomic deep sequencing

**DOI:** 10.1016/j.ajoc.2022.101294

**Published:** 2022-01-20

**Authors:** Miles F. Greenwald, Travis K. Redd, Thuy Doan, Stephen D. McLeod, Gerami D. Seitzman

**Affiliations:** a Department of Ophthalmology, Kellogg Eye Center, University of Michigan Medical Center, 1000 Wall St, Ann Arbor, MI, 48105, United States; b Department of Ophthalmology, Casey Eye Institute, Oregon Health and Science University, 515 SW Campus Dr, Portland, OR, 97239, United States; c Francis I. Proctor Foundation, University of California San Francisco, 490 Illinois St, San Francisco, CA, 94158, United States; d Department of Ophthalmology, University of California San Francisco, 490 Illinois St, San Francisco, CA, 94158, United States

**Keywords:** LASIK Fungal keratitis, LASIK Flap amputation, Metagenomic deep sequencing

## Abstract

**Purpose:**

To describe a unique case of LASIK flap fungal keratitis confirmed by next generation sequencing.

**Observations:**

A 56-year-old female presented with refractory keratitis involving her LASIK flap 21 years after surgery. Confocal was positive for filamentous structures. The patient underwent immediate flap amputation followed by topical antifungal treatment. Corneal culture was positive for *Acremonium* sp. Metagenomic deep sequencing confirmed *Acremonium* as the primary source of infection and also identified *Fusarium* as a likely contributor of a mixed fungal infection. Sequencing also identified hay as the likely source of the infection. Treatment resulted in eradication of the infection. The patient's final best corrected visual acuity was 20/30 with rigid contact lens overrefraction.

**Conclusions:**

Metagenomic deep sequencing is a novel diagnostic tool that is increasingly being utilized for diagnosis of refractory keratitis. This case demonstrates the diagnostic potential of deep sequencing for identifying post-LASIK keratitis and reinforces the utility of LASIK flap amputation in the setting of tectonic flap instability due to keratolysis.

**Importance:**

This case highlights several important clinical points for treating LASIK flap keratitis and highlights the emerging role metagenomic sequencing has in the diagnosis of infectious keratitis. This is first known case using next generation sequencing to diagnose a post-LASIK infectious keratitis.

## 1 Introduction

Infectious keratitis is a rare but potentially vision-threatening complication following LASIK.^1^ Typical onset of post-LASIK infectious keratitis occurs in the days to weeks following surgery, but can occur years later.^1^ Causative organisms are diverse and vary depending on length of time post- LASIK and include *Staphylococcus* and *Streptococcus* in the early postoperative period and atypical bacteria and fungi thereafter.^1^ Infection involving the flap interface requires immediate flap lifting and debridement, with flap amputation required in cases of tectonic flap instability.^1^ Post-LASIK fungal keratitis typically presents more than 2 week postoperatively and often has poor visual outcomes with increased need for flap amputation and/or therapeutic keratoplasty.^2^ Metagenomic deep sequencing is a new diagnostic modality that has been increasingly studied to improve sensitivity of traditional corneal cultures.^3^ We present a case of very late onset LASIK flap fungal keratitis with use of deep sequencing for confirmation of the fungal pathogens.

## 2 Case report

**Fig. 1:**
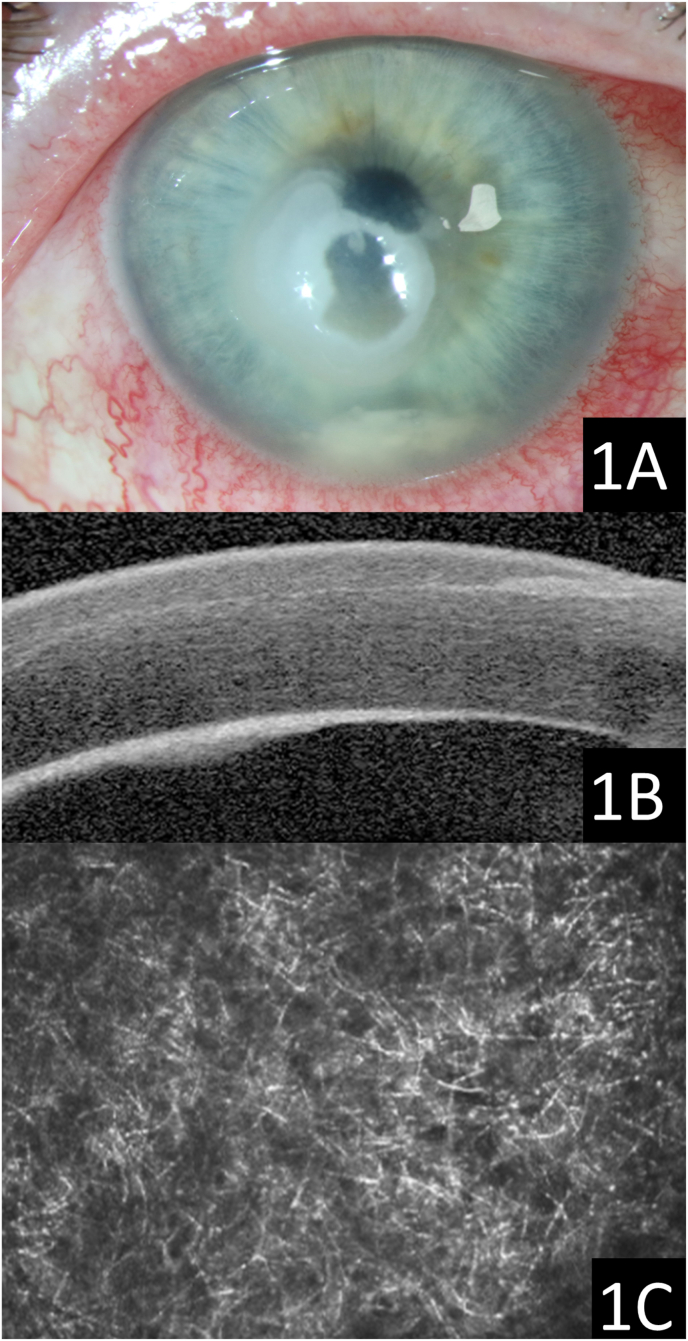
Slit lamp photograph showing dense infiltrate with loculated hypopyon (1A). Anterior segment optical coherence tomography revealing hyperreflectivity in the LASIK flap interface and along the endothelium (1B). Confocal microscopy (depth 55 μm) demonstrating numerous branching filamentous structures consistent with filamentous fungi (1C).

**Fig. 2:**
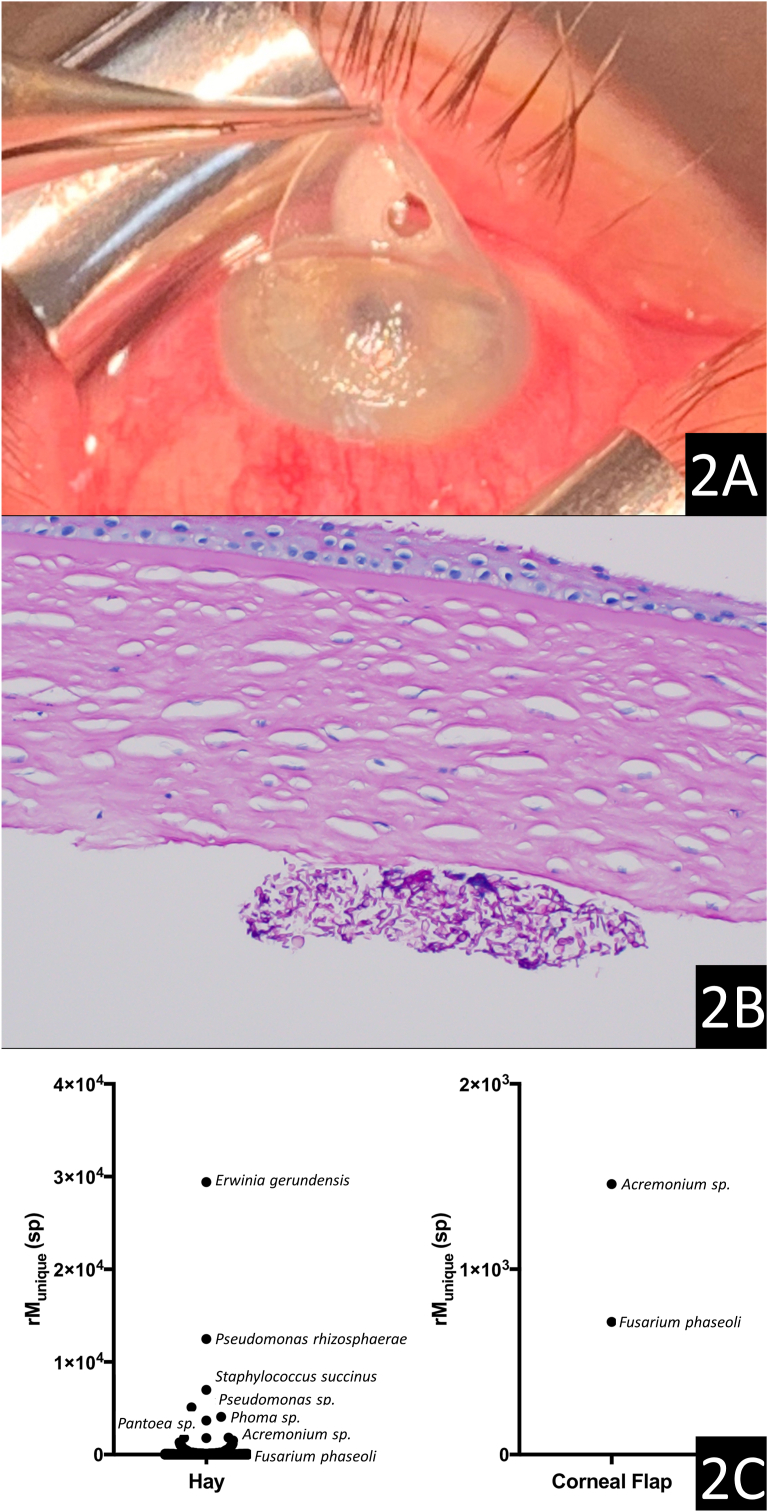
External photo of flap lift with central buttonhole from infectious keratolysis (2A). Surgical pathology evaluation revealing fungal elements on the posterior edge of the amputated flap (2×B) (PAS stain, 20× magnification). Identification of *Acremonium and Fusarium Sp.* from the corneal flap and polymicrobial colonization of hay by metagenomic deep sequencing (MDS). Organisms are plotted as a function of matched read pairs per million read pairs (rM) at the species level based on nucleotide alignment (2C).

A 56-year-old female was referred for a worsening right corneal ulcer. Her past ocular history was significant for laser in situ keratomileusis (LASIK) 21 years prior and right sided soft daily contact lens wear for monovision. Four weeks prior, she presented with right eye pain, redness and decreased vision and was diagnosed with a cornea ulcer. Initial cultures were reportedly negative. She was treated with a variety of topical antibacterial and steroid treatments and the ulcer worsened. On presentation to our referral practice, visual acuity was count fingers and intraocular pressure was normal. Slit lamp examination revealed a dense paracentral chalky white anterior stromal infiltrate with full-thickness involvement of the LASIK flap. The infiltrate extended throughout the flap interface and was associated with central melting resulting in a large buttonhole through the LASIK flap (Fig. 1). An endothelial plaque was noted. Anterior segment optical coherence tomography showed flap interface hyperreflectivity and confirmed the endothelial plaque. Confocal microscopy centered around 55 μm of depth, revealed short branching filaments. The patient underwent immediate flap amputation and hourly topical natamycin was initiated. Cultures revealed *Acremonium* sp., a common agricultural fungus. Next generation sequencing confirmed *Acremonium* sp. infection and identified a second potential pathogen with fewer diagnostic reads, *Fusarium phaesoli.* The patient then revealed she owns a donkey farm and likely shoveled hay that hit her eye. Deep sequencing analysis of a hay sample from this barn identified both pathogens detected in the amputated flap, in addition to over 2000 bacterial and fungal species. The epithelial defect was completely resolved by four weeks after flap amputation. Five months after flap removal, the right cornea healed with a reticular anterior stromal haze. Uncorrected acuity was 20/400. Best corrected vision with rigid lens overrefraction measured 20/30 (see Fig. 2).

## 3 Discussion

We present a unique case of fungal keratitis occurring 21 years following LASIK. *Acremonium* sp. is a genus of filamentous fungi commonly found in soil and decomposing plant material.^4^ *Acremonium* has been rarely reported to cause keratitis, with trauma from vegetable matter being reported in most previous cases.^4^, ^5^, ^6^ Alfonso et al. reported a series of 4 cases with surgeries all conducted in the same operating room found to be contaminated with *Acremonium* on the room's walls and air vents.^6^ *Acremonium* is known to be sensitive to natamycin as was used in our case as well as amphotericin B and voriconazole.^4^ The other fungus confirmed by deep sequencing from the corneal sample was *Fusarium,* a known cause of keratitis.

Metagenomic deep sequencing (MDS) can be used to increase sensitivity of traditional corneal cultures and has been shown to perform especially well in identifying fungal pathogens as was seen in this case.^3^ In addition to confirming the culture results of an atypical organism, MDS also identified a likely secondary pathogen indicating deep sequencing may also be helpful diagnostically in identifying polymicrobial infections. While MDS has clinical utility in identifying atypical corneal pathogens, limitations of this technology include colonization, contamination and sample degradation. To our knowledge, MDS has never before been used to investigate the etiology of post-LASIK keratitis. Given that *Acremonium* and *Fusarium* were both isolated from the corneal sample and the hay and are both known causes of keratitis, it is likely that these fungi represent the etiologic pathogens in this case.

## 4 Conclusion

In summary, we present a case of very late onset post-LASIK *Acremonium* fungal keratitis. Several features of this case are notable: the twenty-one years between surgery and infection, the presumed source of the *Acremonium* fungi from livestock hay and the confirmation of the pathogen and identification of a second pathogen with metagenomic deep sequencing. A key clinical lesson from this case is that LASIK flap amputation should be considered if there is significant flap involvement, loss of tectonic stability due to keratolysis, especially in cases with atypical pathogens.

## Patient consent

Consent to publish the case report was not obtained. This report does not contain any personal information that could lead to the identification of the patient.

## Funding

Both institutions are supported by unrestricted departmental funding from 10.13039/100001818Research to Prevent Blindness (New York, NY).

## Author declaration

All authors report no conflicts of interest pertinent to the case report.

## Intellectual property

We confirm that we have given due consideration to the protection of intellectual property associated with this work and that there are no impediments to publication, including the timing of publication, with respect to intellectual property. In so doing we confirm that we have followed the regulations of our institutions concerning intellectual property.

## Research ethics

We further confirm that any aspect of the work covered in this manuscript that has involved human patients has been conducted with the ethical approval of all relevant bodies and that such approvals are acknowledged within the manuscript.

IRB approval was obtained (required for studies and series of 3 or more cases).

Written consent to publish potentially identifying information, such as details or the case and photographs, was obtained from the patient(s) or their legal guardian(s).

## Declaration of competing interest

No conflict of interest exists.
